# 

**Published:** 1857-01

**Authors:** 


### THE JOURNAL OF PUBLIC HEALTH.

### CONTENTS OF No. VIII.

Induction in the Study of Disease ........................................ 313
Preservation of Food in Ancient and Modern Times ........................ 323

**EPITOME OF SANITARY LITERATURE:**

Contagious Furunculoid Disease ........................................ 336
Preservation of Natural Manures ........................................ *ib.*
Health of the City of London ........................................ 337
Diet in Infantile Eczema ........................................ 338
Treatment of Pulmonary Consumption ........................................ *ib.*
Climate of Aspley Guise ........................................ 342

**ORIGINAL COMMUNICATIONS:**

Protective and Modifying Powers of Vaccination. By E. C. SEATON, M.D. 343

Nursery Government in its Sanitary Aspects. By T. HERBERT BARKER, M.D. (Continued from p. 152) ........................................ 368

Diseases of Colliery Operatives, and their Preventible Causes. By WILLIAM I. COX, ESQ. (Continued from p. 43) ........................................ 374

Notes on certain Vegetable Poisons. By WILLIAM PICKELLS, M.D. ......... 378

**SANITARY AND SOCIAL SCIENCE:**

*Reports on Cities, Towns, and Districts.*—Canterbury ........................ 383

Water Supply of London ........................................ 391

**PROGRESS OF EPIDEMICS in Thirty-eight Towns and Districts** .......... 393

**SANITARY LEGISLATION:**

Drainage of the Metropolis ........................................ 411
Health of Croydon ........................................ *ib.*
Annual Report of the Registrar-General ........................................ 412
Report on Military Prisons ........................................ *ib.*

**TRANSACTIONS of the EPIDEMIOLOGICAL SOCIETY of LONDON:**

Report on Cholera in the Black Sea Fleet. By B. G. BABINGTON, M.D., F.R.S. 89
Quarterly Report of Proceedings ........................................ 116

